# Errors Disrupt Subsequent Early Attentional Processes

**DOI:** 10.1371/journal.pone.0151843

**Published:** 2016-04-06

**Authors:** Liesbet Van der Borght, Hanne Schevernels, Boris Burle, Wim Notebaert

**Affiliations:** 1 Department of Experimental Psychology, Ghent University, Ghent, Belgium; 2 Laboratoire de Neurosciences Cognitives, UMR7291, Aix-Marseille Université, CNRS, Marseille, France; Centre de Neuroscience Cognitive, FRANCE

## Abstract

It has been demonstrated that target detection is impaired following an error in an unrelated flanker task. These findings support the idea that the occurrence or processing of unexpected error-like events interfere with subsequent information processing. In the present study, we investigated the effect of errors on early visual ERP components. We therefore combined a flanker task and a visual discrimination task. Additionally, the intertrial interval between both tasks was manipulated in order to investigate the duration of these negative after-effects. The results of the visual discrimination task indicated that the amplitude of the N1 component, which is related to endogenous attention, was significantly decreased following an error, irrespective of the intertrial interval. Additionally, P3 amplitude was attenuated after an erroneous trial, but only in the long-interval condition. These results indicate that low-level attentional processes are impaired after errors.

## Introduction

Making one error is usually not problematic but it is important to optimize behavior subsequently. For several decades, post-error adaptation has been investigated using behavioural measures with the most robust finding being a slowing in reaction time following an error [1]. Interestingly, this post-error slowing (PES) is reliable within participants when re-tested, suggesting that PES reflects a personal response to the error [2, 3]. PES has been hypothesized to reflect a strategic adaptation in order to reduce the probability of making more errors [4]. Although decreased error rates following errors have been observed [5, 6, 7], other studies have found increased error rates following errors [8, 9, 10]. Therefore, non-functional accounts were postulated which explain PES as a nonspecific result of attention orienting or error processing itself [7, 11]. The orienting account states that errors are infrequent and motivationally salient events that trigger an orienting response [11]. This orienting response interferes with subsequent processing leading to PES and, if the inter-trial interval is short, to a post-error accuracy *decrease*. In line with this idea, Notebaert et al. demonstrated that PES is found when errors are infrequent but post-*correct* slowing is observed when correct responses are infrequent. Similarly, accuracy differences between blocks or participants are reflected in larger PES when less errors are made [10, 12]. A second nonfunctional theory is the bottleneck account [7]. This theory posits that error-monitoring requires time and resources from a capacity-limited cognitive system and therefore interferes with the task at hand. Because of this central bottleneck, responses are slower and more error-prone immediately following an error.

The difference between functional and non-functional accounts lies in the interpretation of the slowing. Functional accounts assume that slowing pertains to performance optimization, while non-functional accounts do not. This difference has typically been investigated by comparing accuracy levels preceding and following errors, where functional accounts predict increased post-error performance while nonfunctional accounts predict decreased post-error performance. This behavioral measure, however, relies on the frequency of double errors (which decreases post-error accuracy and, hence, the support for ‘functionality’). However, the frequency of double errors will always increase with overall error frequency and double errors can occur for various reasons (distraction, confusion of mapping rule, …).

In order to investigate post-error performance without relying on double-errors, Houtman and Notebaert [13] used a speeded flanker task that was followed by a rapid serial visual presentation (RSVP) task in which participants had to detect a letter in a rapid presentation of a series of numbers. Houtman and Notebaert showed decreased target detection following errors in the flanker task. In the present study, we compare visually evoked electrophysiological components following errors and correct trials. We present a speeded flanker task followed by a visual discrimination task with one frequent and one infrequent target (triangle or diamond). We hypothesized that the early visual components (P1, N1) as well as the P3, which is typically related to the updating of working memory [14], would be attenuated following an error. We had a short and a long interval (between-subjects) between both tasks. The effects should be more pronounced in the short-interval condition than in the long-interval condition. Additionally, error-related components, such as the error-related negativity (ERN) and subsequent the early and late positive deflections (Pe), related to the flanker task were investigated.

## Method

### Participants

Thirty healthy right-handed participants with normal vision or corrected-to-normal vision participated in the experiment (three male; mean age 20 years, range 18–23 years). All participants were neurologically and psychiatrically healthy and each gave written informed consent. The study was approved by the ethical committee of the Faculty of Psychology and Educational Sciences of Ghent University. Participants were compensated at 15€ per hour.

### Stimuli and Procedure

A flanker task and a visual discrimination task were combined to investigate early visual ERP components. Stimuli of the flanker task consisted of four possible letters: H, S, X, Z. In the flanker task participants had to respond by pressing a button with the index or middle finger of the right hand according to the identity of the central letter. Two letters were (randomly) mapped on each button resulting in six possible response mappings which where counterbalanced over the participants. In the visual discrimination task, a response had to be given with the index or middle finger of the left hand. Two possible figures were used, a diamond or a triangle, which were presented with either 80% or 20% frequency. Which figure was presented infrequently, i.e. in 20% of the trials, was counterbalanced over participants. There were two possible response mappings for the frequency task, which was also counterbalanced over participants.

The participants were seated in a comfortable armchair in a light-dimmed and sound-attenuated room. They were tested on a Pentium IV personal computer with a 17-inch monitor running Tscope [15]. Participants gave a manual response with the left and right index fingers of both hands using a Cedrus response box. Stimuli were presented centrally in white on a black background. A trial started with the presentation of the stimuli of the flanker task for 100 ms. Next, a mask was presented (#####) for 150 ms followed by a blank screen until a response was given or the response deadline was exceeded (750 ms from stimulus onset). Subsequently, a blank screen was presented for 500 ms for one group of participants (i.e. short interval) or 1000 ms for the other group of participants (i.e. long interval). Accordingly, the visual discrimination task started with the presentation of a figure (diamond or triangle) during 500 ms followed by a blank screen with a duration of 500 ms. When a response was given within the response deadline of 1000 ms the stimuli disappeared and a blank screen was presented for the remaining time. At the end of each trial, a fixation stimulus was displayed (+) for a randomly selected time interval of 200 to 500 ms.

Participants were asked to refrain from blinking during trials. The response mapping for both tasks was explained followed by a practice block for both tasks separately. Each practice session included 140 trials and feedback informed participants about their performance. Subsequently, the experiment consisting of 16 runs of 70 trials, was performed. During each break response mappings were repeated and overall performance during the last block was shown (i.e. accuracy levels and the percentage of too slow responses in both tasks). When participants gave more than 10% too slow responses, an additional message was shown to encourage participants to respond faster.

### EEG acquisition and preprocessing

We recorded EEG activity with a Biosemi ActiveTwo measurement system (BioSemi, Amsterdam, Netherlands) with scalp electrodes (64 Ag-AgCl attached in an elastic cap) arranged according to the standard international 10–20 system. Additionally, five external electrodes were attached to the head: left and right mastoid, which were used for later offline re-referencing, beneath the right eye and a bilateral electro-oculogram (EOG) electrode pair next to the outer canthi of the eyes referenced to each other to measure horizontal eye movements. Signals were amplified and digitized with a sampling rate of 512 Hz. EEG data was processed using EEGLAB and the ERPLAB plugin (http://erpinfo.org/erplab), running on MATLAB. Data of one electrode (TP7) was removed for all participants since this electrode malfunctioned in more than half of the participants. Independent component analysis (ICA) was conducted to identify and remove stereotypical eye blink components. In order to explore visual components in the visual discrimination task, epochs were created time-locked to the onset of the stimulus of the frequency task, including a 200 ms pre-stimulus period that was used for baseline correction and a 800 ms post-stimulus interval. Hence, the total time window of these epoched ERPs was 1000 ms. In order to investigate error-related components; epochs were created locked to the response on the flanker task, starting from 400 ms before response onset until 1600 ms after response onset. The epochs were baseline-corrected using the 400 to 200 ms pre-stimulus window. Additional EMG activity per epoch was removed with blind source separation (BSS) using the AAR toolbox (http://www.germangh.com/eeglab_plugin_aar/index.html). As in a previous study, we used Laplacian transformation, which allows spatial deblurring of EEG [16], to dissociate the ERN/CRN [17, 18] and the early from the late Pe-component [19]. Furthermore, Laplacian transformation also improves the spatial and temporal resolution of visual evoked potentials [20–22]. Given that the use of Laplacian transformation is very sensitive to local artifacts, epochs were manually inspected and rejected if necessary. On average respectively 10 and 8% of the epochs were excluded. EEG epochs were averaged across participants according to the different conditions. Infrequent targets following erroneous responses (least frequent cells) were calculated on an average of 38 trials (*SD* = 21) per participant. Only 2 participants had less than 10 observations in this cell. Excluding these participants did not change the results in a significant way. The monopolar averages were then transformed using the CSD toolbox for Laplacian transformation [23], http://psychophysiology.cpmc.columbia.edu/software/CSDtoolbox), thereby enhancing the spatial resolution and intensity of ERP components. Current source densities (CSDs) were calculated according to the spherical spline algorithm [24], using a default smoothing constant of 1.0^−5^ and a head radius of 10 cm. Note that transformation via CSDs results in reference-free ERP data

### EEG analyses

Mean amplitudes were derived over a number of electrodes within a certain time-window as defined by previous literature and inspected on ERP waveforms and topographic maps collapsed across conditions. For error-related components, the ERN and CRN were measured at electrode FCz in a time window between 0 and 100 ms after response execution in the flanker task. As reported in the literature, the Pe consisted of two subcomponents [19, 25, 26]; one early component maximal between 80 and 180 ms at Cz, and a later broader component measured between 300 and 500 ms at POz.

In the visual discrimination task we analyzed the stimulus-locked P1 component which was quantified at posterior electrodes PO7 and PO8 between 80 and 130 ms. This component was followed by a negative wave (N1) over the same electrodes from 130 to 180 ms. Furthermore, the P3 was quantified at Pz between 400 and 600 ms.

Amplitudes for error-related components were examined using a repeated-measures analysis of variance (rANOVA) with the between-subjects factor interval condition (short or long) and the within-subjects factor previous accuracy (correct, error). In relation to stimulus-locked components, the between-subjects factor interval condition and the within-subjects factors previous accuracy (in the flanker task) and stimulus frequency (frequent, infrequent) of the visual discrimination task, were included. Trials with exceptionally fast (< 100 ms) or no responses were excluded (i.e., exceeding the response deadline), since it is unclear whether the stimulus was perceived in this case. Additionally, we used the Pearson correlation coefficient (*r*) to examine the relationship between early visual components and error-related components as well as behavioural measures.

## Results

### Flanker task

On average, participants responded correctly in 80% (*SD* = 11%) of the trials in the flanker task and the average correct reaction time was 589 ms (*SD* = 71 ms). Furthermore, we did not observe a significant difference between both interval conditions, both *ps* ≥ 0.54.

### Visual discrimination task

#### Reaction times

**Fig 1:**
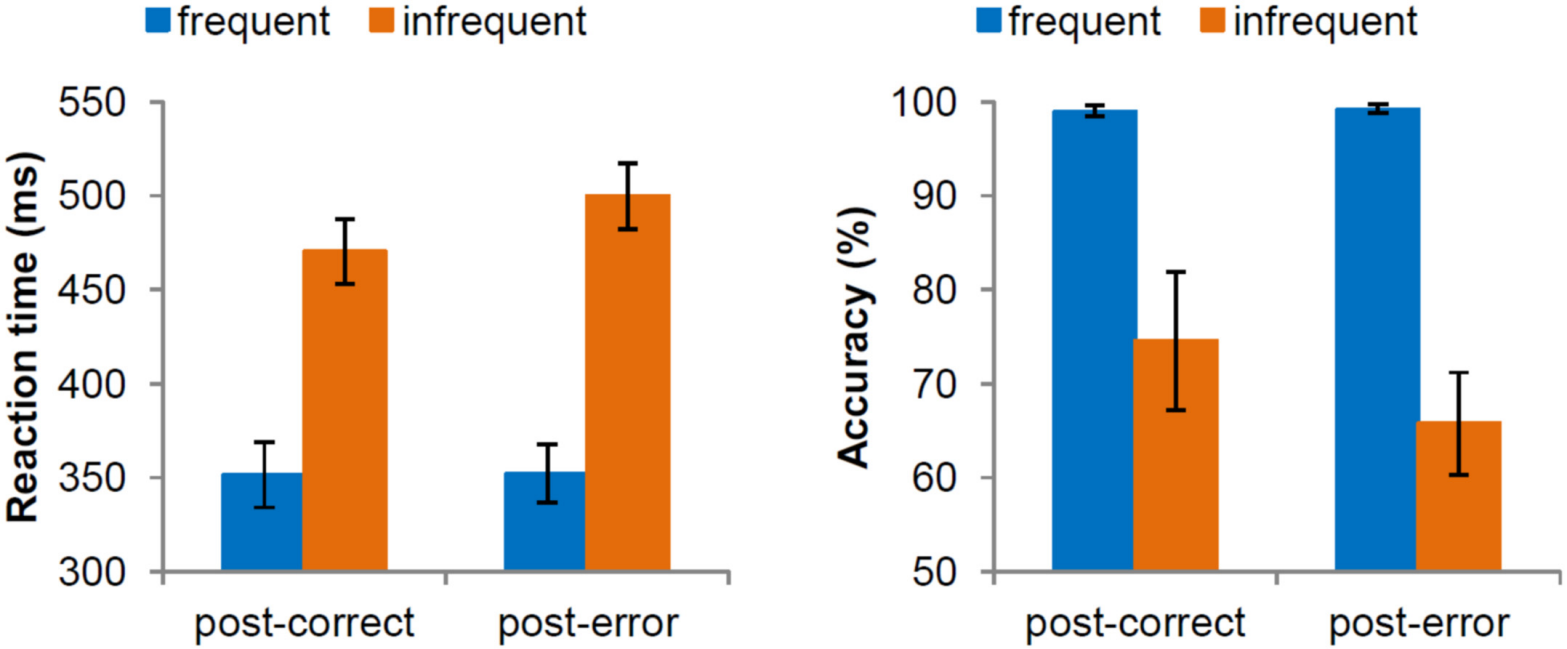
Average reaction time (in millisecondes) and accuracy (in percentages) in trials with frequent and infrequent stimuli following correct and erroneous trials in the flanker task. Error bars represent 95% confidence intervals around the means.

There was no significant difference between interval conditions, *F*(1,28) = 1.43, *p* = 0.24. Yet, we did detect a significant effect of target frequency, *F*(1,28) = 467.88, *p* < 0.001, showing slower responses for an infrequent target (485 ms) than for a frequent target (352 ms). There was also a main effect of previous accuracy in the flanker task, *F*(1,28) = 14.59, *p* = 0.001, indicating post-error slowing (15 ms). The interaction of frequency and previous accuracy in the flanker task also reached significant levels, *F*(1,28) = 22.06, *p* < 0.001 (Fig 1). More specifically, reaction times for frequent stimuli did not depend on previous accuracy, *F*(1,28) = 0.04, *p* = 0.84, while reaction time for infrequent stimuli was significantly slower following an error than following a correct response in the flanker task, *F*(1,28) = 25.45, *p* < 0.001. Additionally, the interaction of previous accuracy in the flanker task and interval was marginally significant, *F*(1,28) = 3.17, *p* = 0.08, showing larger PES in the short interval condition (22 ms) compared to the long interval condition (8 ms). All other interactions were not significant *p* ≥ 0.63.

#### Accuracy

We did not find a significant difference between interval conditions, *F*(1,28) = 0.20, *p* = 0.66. However, there was a significant effect of frequency, *F*(1,28) = 99.21, *p* < 0.001, with impaired performance in trials with infrequent targets (70%) compared to frequent targets (99%). Also, a main effect of previous accuracy in the flanker task was detected, *F*(1,28) = 12.97, *p* = 0.001, showing post-error decrease in accuracy (-4%). Interestingly, the interaction of frequency and previous accuracy in the flanker task was also significant, *F*(1,28) = 14.25, *p* = 0.001. Specifically, for frequent targets there was no influence of previous accuracy, *F*(1,28) = 0.44, *p* = 0.51, yet performance in trials with infrequent targets was significantly reduced following an error, (66%) than following a correct response (75%), *F*(1,28) = 13.66, *p* = 0.001. All other interactions were not significant *p* ≥ 0.67.

### Error-related components on the flanker task

**Fig 2:**
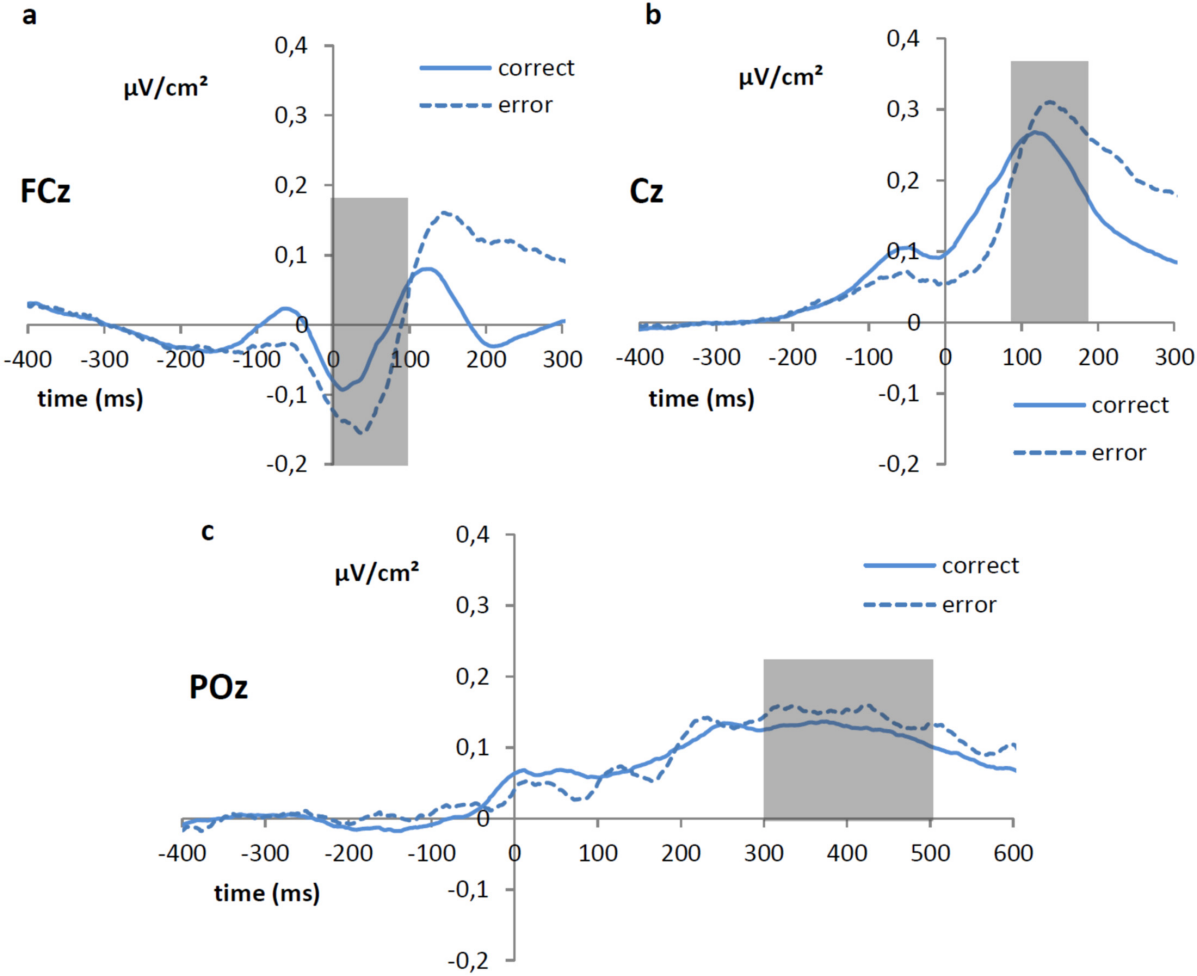
Laplacian transformed grand average (in μV/cm^2^) response-locked ERP waveforms showing the (a) ERN/CRN (measured between 0 and 100 ms at Fcz), (b) the early Pe/Pc (measured between 80 and 180 ms at Cz) and (c) the late Pe/Pc (measured between 300 and 500 ms at POz) as a function of current accuracy.

Related to the mean amplitude of the ERN and CRN, no significant main effect of the factor interval condition was observed, *F*(1,28) = 0.58, *p* = 0.45. However, a main effect of accuracy on the current trial was found, *F*(1,28) = 14.22, *p* = 0.001, with the mean amplitude of the ERN (Fig 2a) being more negative (-0.10 μV/cm^2^) compared to the mean amplitude of the CRN (-0.04 μV/cm^2^). The interaction between the factors interval condition and accuracy of the current response was not significant, *F*(1,28) = 0.98, *p* = 0.33.

When turning to the early Pe/Pc components (Fig 2b), no significant main effect of interval condition was detected, *F*(1, 28) = 0.24, *p* = 0.63. There was a significant effect of accuracy, *F*(1, 28) = 7.79, *p <* 0.01, with the mean amplitude being more positive in trials with an erroneous (0.28 μV/cm^2^) than a correct response (0.24 μV/cm^2^). Moreover, the interaction between the factors interval condition and accuracy did not reach significance, *F*(1, 28) = 2.03, *p* = 0.17.

The mean amplitude of the late Pe/Pc (Fig 2c) again did not show a significant effect of interval condition, *F*(1, 28) = 0.51, *p* = 0.48. Also, there was no significant effect of accuracy, *F*(1, 28) = 2.68, *p* = 0.11, nor a significant interaction between interval condition and previous accuracy, *F*(1, 28) = 0.26, *p* = 0.61.

### Stimulus-related components in the visual discrimination task

Results indicated a significant main effect of frequency on **P1 mean amplitude**, *F*(1,28) = 6.06, *p* = 0.02, showing more positive values for trials with frequent stimuli (0.19 μv/cm^2^) than for trials with infrequent stimuli (0.16 μv/cm^2^). No other significant effects were observed, all *ps* ≥ 0.09.

**Fig 3:**
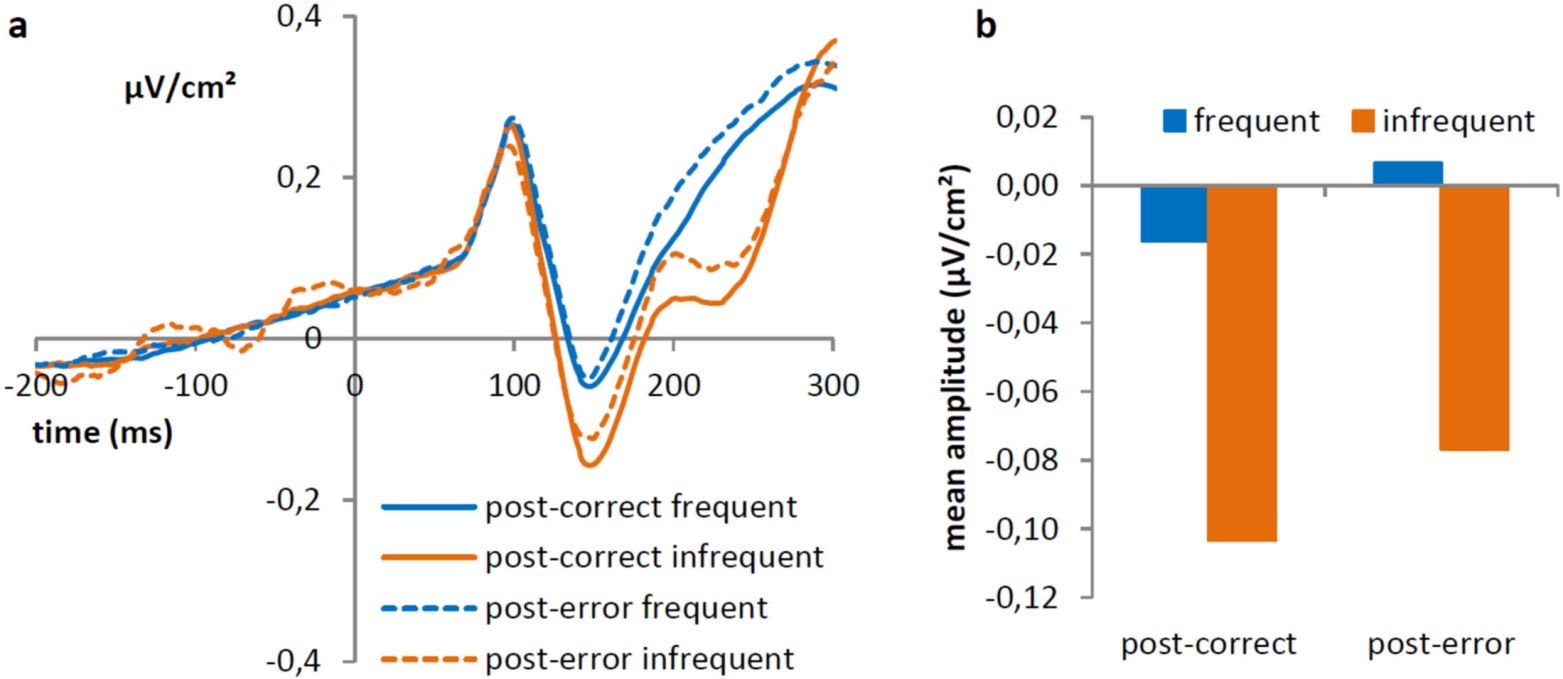
Left side of the figure (a) shows the Laplacian transformed grand average ERP waveforms at PO7 and PO8 showing the P1 (measured between 80 and 130 ms) and N1 (measured between 130 and 180 ms) as a function of previous accuracy in the flanker task and frequency. On the right side (b) average amplitude (in μV/cm^2^) of the N1, for frequent and infrequent stimuli following correct and erroneous trials in the flanker task are shown.

For to the **N1 mean amplitude**, we observed a significant effect of interval condition, *F*(1,28) = 7.71, *p* = 0.01, showing more negative values in the long interval condition (-0.16 μv/cm^2^) then in the short interval condition (0.07 μv/cm^2^). Furthermore, there was a significant effect of frequency, *F*(1,28) = 23.15, *p* < 0.001, showing that trials with infrequent stimuli show increased negative amplitudes (-0.09 μv/cm^2^) compared to trials with frequent stimulus (-0.01 μv/cm^2^). There was also a significant effect of previous accuracy, *F*(1,28) = 5.06, *p* = 0.03, showing more negative values following a correct (-0.06 μv/cm^2^) compared to an erroneous response (-0.04 μv/cm^2^), Fig 3. The interaction between previous accuracy and frequency was not significant, *F*(1,28) = 0.09, *p* = 0.76, as were the interactions with the factor interval condition, all *ps* ≥ 0.62.

**Fig 4:**
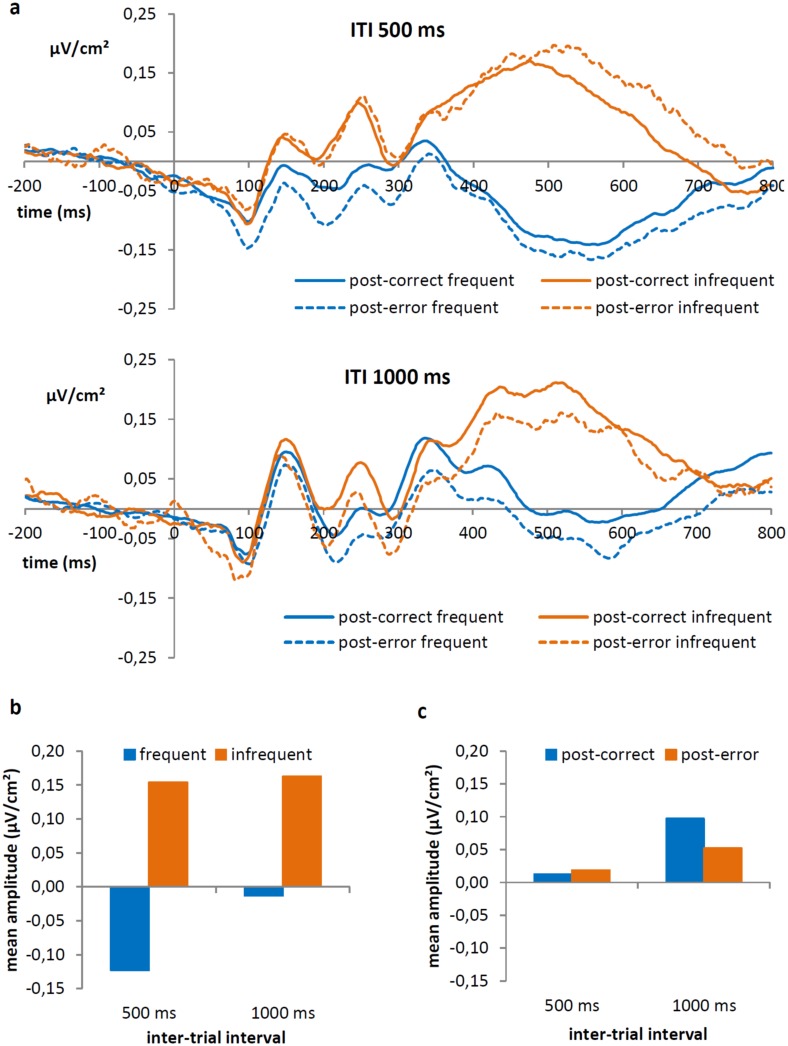
(a) Laplacian transformed grand average ERP waveforms at Pz, measured between 400 and 600 ms, as a function of previous accuracy in the flanker task and frequency, separately for interval condition. (b) Average P3 amplitudes (in μV/cm^2^) for frequent and infrequent stimuli in both conditions are shown. (c) Average amplitude (in μV/cm^2^) of the P3, for post-correct and post-error trials in both conditions.

We detected a significant main effect of frequency on **P3 amplitudes**, *F*(1,28) = 92.86, *p* < 0.001. As clearly illustrated in Fig 4a, positive values were observed for infrequent stimuli (0.16 μv/cm^2^) but not for frequent stimuli (-0.07 μv/cm^2^). Interestingly, this effect interacted with interval condition, *F*(1,28) = 4.71, *p* = 0.04 (Fig 4b), showing that the difference between frequent and infrequent targets was larger in the short interval condition (0.28 μV/cm^2^, frequent: -0.12 μV/cm^2^, infrequent: 16μV/cm^2^) than in the long interval condition (0.18 μV/cm^2^, frequent: -0.01 μV/cm^2^, infrequent: 16μV/cm^2^). Furthermore, there was no significant main effect of interval condition or previous accuracy in the flanker task, both *ps* ≥ 0.16, but there was a marginally significant interaction between the factors interval condition and previous accuracy in the flanker task, *F*(1,28) = 3.68, *p* = 0.07 (Fig 4). Specifically, in the short condition the effect of previous accuracy was not significant (post-correct: 0.01 μV/cm^2^, post-error: 0.02 μV/cm^2^), *F*(1,28) = 0.10, *p* = 0.75. In the long condition however, amplitudes following a correct response (0.10 μV/cm^2^) were significantly higher than following an error (0.05 μV/cm^2^), *F*(1,28) = 5.71, *p* = 0.03 (Fig 4c). Results indicated no other significant main effects or interactions, all *ps* ≥ 0.21.

### Correlations

We did not observe a significant correlation between any of the error-related components during the flanker task and the effects in the visual discrimination task, all *ps* ≥ 0.25. Moreover, the N1 effect did not correlate significantly with any of the behavioural measures, all *ps* ≥ 0.50.

The P3 effect in the long RSI condition correlated significantly with the effect on accuracy, *r*(15) = 0.52, *p* < 0.05, and marginally significant with PES, *r*(15) = 0.49, *p* = 0.07, indicating that participants with a larger P3 amplitude following an error (compared to a correct response), showed less post-error accuracy decrease and larger PES. However, deleting two online outliers rendered both correlations non-significant, resp. *r*(13) = -0.01, *p* = 0.98 and *r*(13) = 0.17, *p* = 0.57. There were no significant correlations in the short interval condition, all *ps* ≥ 0.49.

## General Discussion

In this experiment, we investigated the effect of errors on subsequent visuo-attentional processes by combining a flanker and a visual discrimination task. Additionally, the intertrial interval between both tasks was manipulated in order to explore the duration of these negative after-effects. As previously reported, the ERN/CRN was followed by an early and late Pe/Pc in the flanker task [19, 25, 26]. While the early Pe/Pc was significantly larger for errors, there was no significant difference in late Pe/Pc amplitude between erroneous and correct trials. Since the late Pe/Pc is believed to be related to error awareness [25, 27, 28], it is possible that participants did not consciously know when they made an error. The amplitude of the N1 in the visual discrimination task was significantly decreased after an erroneous compared to a correct response in the flanker task, irrespective of the intertrial interval, suggesting diminished visual attention to the task-relevant stimulus after an error. Furthermore, P3 amplitude was also attenuated following an error, but only in the long condition, showing impaired memory updating following an error.

After committing an error in the flanker task, the N1 but not the P1 amplitude locked to the target of the visual discrimination task was attenuated. This is in line with the idea that the P1 represents early sensory processing in a location where attention is already focused while the N1 rather reflects the orienting of attention to task-relevant stimuli [29, 30]. Because the stimulus of the visual discrimination task was presented in the center of the screen, as were the stimuli in the flanker task, it is not surprising that bottom-up attention (reflected in the P1) is not influenced. However, infrequent stimuli did elicit a smaller P1 component, indicating that bottom-up attention is suppressed when more top-down attention is recruited, as illustrated by a larger N1 amplitude for infrequent stimuli.

The reduced N1 following an error illustrates that participants have less resources to actively focus attention on the stimulus of the visual discrimination task, irrespective of frequency. Interestingly, only performance on infrequent stimuli was impaired following an error. This suggests that frequent stimuli might have been processed rather automatically and with a minimum of resources, while the processing of infrequent stimuli did suffer from decreased attentional resources.

While an attentional blink-like effect can be found following errors [13], our results show that the mechanism behind the classic attentional blink and the error-induced blink are most likely not the same since previous research showed that only P3 amplitude, and not the N1, is attenuated in classical AB tasks [31, 32] suggesting an impairment in memory updating. However, similar to the idea that an emotional attentional blink occurs through competition for perceptual resources [33, 34], our results show that an error is followed by a decrease in attentional resources, which is generally in line with the orienting account [11]. Our findings are more difficult to align with the bottleneck account as the bottleneck occurs on central processing stages but not on perceptual stages [7]. The bottleneck would therefore predict similar results as found for the classic attentional blink effect.

Taken together, our results show that visual attention following an error, as indexed by the N1 component, is attenuated. This suggests that attention is directed away from the task, presumable due to an error-related orienting response [11]. Additionally, memory-updating, as indexed by the P3 component is reduced following an error, implying that the initial attenuation of attention might also influence higher order processing.

## Supporting Information

S1 Dataset Raw Behavioral Data. (CSV)

S2 Dataset ERP Laplacian Transformed Stimulus Locked. (ZIP)

S3 Dataset ERP Laplacian Transformed Response Locked. (ZIP)

## References

[1] Rabbitt P (1979) How old and young subjects monitor and control responses for accuracy and speed. British Journal of Psychology, 70(2), 305–311.

[2] Danielmeier C, Ullsperger M (2011) Post-error adjustments. Frontiers in Psychology, 2, 233 10.3389/fpsyg.2011.00233 21954390 PMC3173829

[3] Segalowitz SJ, Santesso DL, Murphy TI, Homan D, Chantziantoniou DK, Khan S (2010) Retest reliability of medial frontal negativities during performance monitoring. Psychophysiology, 47(2), 260–70. 10.1111/j.1469-8986.2009.00942.x 20030755

[4] Botvinick MM, Braver TS, Barch DM, Carter CS, Cohen JD (2001) Conflict monitoring and cognitive control. Psychological Review, 108(3), 624–652. 10.1037/0033-295X.108.3.624 11488380

[5] Danielmeier C, Eichele T, Forstmann BU, Tittgemeyer M, Ullsperger M (2011) Posterior medial frontal cortex activity predicts post-error adaptations in task-related visual and motor areas. The Journal of Neuroscience, 31(5), 1780–1789. 10.1523/JNEUROSCI.4299-10.2011 21289188 PMC6623722

[6] Maier ME, Yeung N, Steinhauser M (2011) Error-related brain activity and adjustments of selective attention following errors. NeuroImage, 56, 2339–2347. 10.1016/j.neuroimage.2011.03.083 21511043

[7] Jentzsch I, Dudschig C (2009) Why do we slow down after an error? Mechanisms underlying the effects of posterror slowing. Quarterly Journal of Experimental Psychology, 62(2), 209–218. 10.1080/17470210802240655 18720281

[8] Bombeke K, Schouppe N, Duthoo W, Notebaert W (2013) The effect of alcohol and placebo on post-error adjustments. Frontiers in Human Neuroscience, 7(3). 10.3389/fnhum.2013.00003 PMC3555120 23355819

[9] Carp J, Compton RJ (2009) Alpha power is influenced by performance errors. Psychophysiology, 46, 336–343. 10.1111/j.1469-8986.2008.00773.x 19207203

[10] Houtman F, Núñez Castellar E, Notebaert W (2012) Orienting to errors with and without immediate feedback. Journal of Cognitive Psychology, 24(3), 278–285. 10.1080/20445911.2011.617301

[11] Notebaert W, Houtman F, Van Opstal F, Gevers W, Fias W, Verguts T (2009) Post-error slowing: an orienting account. Cognition, 111, 275–279. 10.1016/j.cognition.2009.02.002 19285310

[12] Steinborn MB, Flehmig HC, Bratzke D, Schröter H (2012) Error reactivity in self-paced performance: Highly-accurate individuals exhibit largest post-error slowing. Quarterly Journal of Experimental Psychology, 65(4), 624–31. 10.1080/17470218.2012.660962 22463389

[13] Houtman F, Notebaert W (2013) Blinded by an error. Cognition, 128(2), 228–236. 10.1016/j.cognition.2013.04.003 23688649

[14] Polich J (2007) Updating P300: an integrative theory of P3a and P3b. Clinical neurophysiology, 118(10), 2128–2148. 17573239 10.1016/j.clinph.2007.04.019 PMC2715154

[15] Stevens M, Lammertyn J, Verbruggen F, Vandierendonck A (2006) Tscope: A C library for programming cognitive experiments on the MS Windows platform. Behavior Research Methods, 38(2), 280–286. 10.3758/BF03192779 16956104

[16] Babiloni F, Cincotti F, Carducci F, Rossini PM, Babiloni C (2001) Spatial enhancement of EEG data by surface Laplacian estimation: the use of magnetic resonance imaging-based head models. Clinical Neurophysiology, 112(5), 724–727. 10.1016/S1388-2457(01)00494-1 11336885

[17] Allain S, Carbonnell L, Falkenstein M, Burle B, Vidal F (2004) The modulation of the Ne-like wave on correct responses foreshadows errors. Neuroscience Letters, 372(1–2), 161–6. 10.1016/j.neulet.2004.09.036 15531109

[18] Vidal F, Hasbroucq T, Grapperon J, Bonnet M (2000) Is the "error negativity" specific to errors? Biological Psychology, 51(2–3), 109–128. 10.1016/S0301-0511(99)00032-0 10686362

[19] Van der Borght L, Houtman F, Burle B, Notebaert W (2016) Distinguishing the influence of task difficulty on error-related ERPs using surface Laplacian transformation. Biological psychology, 115, 78–85. 10.1016/j.biopsycho.2016.01.013 26829762

[20] Burle B, Spieser L, Roger C, Casini L, Hasbroucq T, Vidal F (2015) Spatial and temporal resolutions of EEG: Is it really black and white? A scalp current density view. International Journal of Psychophysiology?: Official Journal of the International Organization of Psychophysiology. 10.1016/j.ijpsycho.2015.05.004 PMC4548479 25979156

[21] Fahrenfort JJ, Scholte HS, Lamme VaF (2007) Masking Disrupts Reentrant Processing in Human Visual Cortex. Journal of Cognitive Neuroscience, 19(9), 1488–1497. 10.1162/jocn.2007.19.9.1488 17714010

[22] Riès S, Janssen N, Burle B, Alario F (2013) Response-locked brain dynamics of word production. PloS One, 8(3). 10.1371/journal.pone.0058197 PMC3595260 23554876

[23] Kayser J, Tenke CE (2006) Principal components analysis of Laplacian waveforms as a generic method for identifying ERP generator patterns: I. Evaluation with auditory oddball tasks. Clinical Neurophysiology, 117(2), 348–368. 10.1016/j.clinph.2005.08.034 16356767

[24] Perrin F, Pernier J, Bertrand O, Echallier JF (1989) Spherical splines for scalp potential and current density mapping. Electroencephalography and Clinical Neurophysiology, 72(2), 184–187. 10.1016/0013-4694(89)90180-6 2464490

[25] Endrass T, Reuter B, Kathmann N (2007) ERP correlates of conscious error recognition: aware and unaware errors in an antisaccade task. The European Journal of Neuroscience, 26(6), 1714–20. 10.1111/j.1460-9568.2007.05785.x 17880402

[26] Van Veen V, Carter CS (2002) The timing of action-monitoring processes in the anterior cingulate cortex. Journal of Cognitive Neuroscience, 14(4), 593–602. 10.1162/08989290260045837 12126500

[27] Shalgi S, Barkan I, Deouell LY (2009) On the positive side of error processing: error-awareness positivity revisited. The European Journal of Neuroscience, 29(7), 1522–32. 10.1111/j.1460-9568.2009.06690.x 19519632

[28] Wessel JR, Danielmeier C, Ullsperger M (2011) Error awareness revisited: accumulation of multimodal evidence from central and autonomic nervous systems. Journal of Cognitive Neuroscience, 23(10), 3021–3036. 10.1162/jocn.2011.21635 21268673

[29] Hopfinger JB, West VM (2006) Interactions between endogenous and exogenous attention on cortical visual processing. NeuroImage, 31(2), 774–89. 10.1016/j.neuroimage.2005.12.049 16490366

[30] Luck S, Heinze H, Mangun G, Hillyard S (1990) Visual event-related potentials index focused attention within bilateral stimulus arrays. II. Functional dissociation of P1 and N1 components. Electroencephalography and Clinical Neurophysiology, 75, 528–542. Retrieved from http://www.sciencedirect.com/science/article/pii/001346949090139B 1693897 10.1016/0013-4694(90)90139-b

[31] Sergent C, Baillet S, Dehaene S (2005) Timing of the brain events underlying access to consciousness during the attentional blink. Nature Neuroscience, 8(10), 1391–400. 10.1038/nn1549 16158062

[32] Vogel EK, Luck SJ, Chun M, Di Lollo V, Egeth H, Maki B, Raymond J (1998) Electrophysiological Evidence for a Postperceptual Locus of Suppression During the Attentional Blink, 24(6), 1656–1674. 10.1037//0096-1523.24.6.1656 9861716

[33] Most SB, Wang L (2011) Dissociating spatial attention and awareness in emotion-induced blindness. Psychological Science, 22(3), 300–5. 10.1177/0956797610397665 21270446

[34] Wang L, Kennedy BL, Most SB (2012) When emotion blinds: a spatiotemporal competition account of emotion-induced blindness. Frontiers in Psychology, 3(November), 438 10.3389/fpsyg.2012.00438 23162497 PMC3491583

